# Adverse childhood experiences, mental illness, HIV and offending among female inmates in Durban, South Africa

**DOI:** 10.4102/sajpsychiatry.v30i0.2108

**Published:** 2024-01-24

**Authors:** Samantha Naidoo, Saeeda Paruk, Liezel Ferreira, Ugasvaree Subramaney

**Affiliations:** 1 Department of Psychiatry, Faculty of Health Sciences, University of the Witwatersrand, Johannesburg, South Africa; 2 Discipline of Psychiatry, College of Health Sciences, University of KwaZulu-Natal, Durban, South Africa

**Keywords:** adverse childhood experiences, mental illness, violent offending, HIV, female inmates, South Africa

## Abstract

**Background:**

Childhood adversities and adult trauma are common among female inmates. Associations have been documented with childhood adversities and mental illness, personality disorders, human immunodeficiency virus (HIV) and violent offending. However, no such study had been conducted in South Africa (SA), despite the high prevalence of HIV and trauma in SA.

**Aim:**

To measure the prevalence of childhood adversities and adult trauma; and to determine if there is a relationship between childhood adversities, mental illness, personality disorders, HIV and violent offending among female inmates.

**Setting:**

The study was conducted at the largest correctional centre in Durban, KwaZulu-Natal, South Africa.

**Methods:**

This cross-sectional, descriptive study randomly recruited 126 female inmates. The World Health Organization’s Adverse Childhood Experiences- International Questionnaire (WHO ACE-IQ) was used to measure childhood adversities; the Structured Clinical Interview for the Diagnostics and Statistical Manual-5 Research Version (SCID 5-RV) was used to diagnose mental illness; and a structured questionnaire was used to measure adult trauma. Human immunodeficiency virus data was confirmed from prison medical records.

**Results:**

Elevated rates of individual childhood adversities and adult trauma were found. Associations were found between cumulative childhood adversities and post-traumatic stress disorder (PTSD), alcohol use disorder, substance use disorder, borderline personality disorder, and HIV.

**Conclusion:**

Female inmates are a highly traumatised population. Prison mental health services should provide trauma-informed and trauma-focussed care to improve inmates’ mental health outcomes and decrease recidivism.

**Contribution:**

This study contributes to the emerging literature on adverse childhood experiences (ACEs) and their associations among incarcerated female populations, in a low- and middle-income, South African setting.

## Introduction

Early childhood is a critical period of development, with rapid physiological, psychological, and cognitive changes.^1^ Trauma in early childhood can result in lifelong impairments in health.^2^ Stressful experiences during this period can result in alterations in brain structure and function, and may have a detrimental effect on the brain’s reactivity to stress.^3,4^ Adverse childhood experiences (ACEs) are defined as ‘traumatic occurrences before the age of 18 years that are experienced as physically or emotionally harmful or threatening’.^5^ They include maltreatment, neglect, household dysfunction and environmental influences, such as living with a household member who abuses substances, or witnessing violence towards a household member.^6^

### Adverse childhood experiences and mental illnesses in the general and prison populations

Literature suggests an association between ACEs and mental health outcomes.^7^

The ACE study, which was a seminal study in the United States of America (US) during the 1990s, resulted in the Centres for Disease Control and Prevention (CDC) and Kaiser Permanente developing the 10-item ACE scale.^8^ Using this scale, they found a high prevalence of ACEs in the general population and a dose-response relationship between childhood adversity and health risk behaviours, which may ultimately contribute to morbidity and mortality.^7^ In the ACE study, 52% of the sample had one or more ACEs, while 6.2% had four or more ACEs.^7^

The prevalence of ACEs differs from one country to another, but high prevalence rates have been found globally. While studies on ACEs are limited in low- and middle-income countries (LMICs) compared to high income countries (HICs),^9^ there is still substantive literature on ACEs in LMICs.^10,11^ One of the limited studies in the South African general population found higher rates of adversities in the general population compared to HICs on a 13-item modified CDC-Kaiser Permanente ACE scale.^12^

One population at increased risk of having experienced ACEs, is prisoners.^13,14^ It has consistently been documented that many prisoners have experienced multiple types of trauma during childhood and adulthood.^15,16^ A systematic review examining ACEs and subsequent psychopathology in prisoners, with studies predominantly from the US and Europe, confirmed an association between childhood adverse events and adult psychiatric disorders.^17^ However, it did not contain any studies from low-income countries and specifically, none from Africa. Studies of ACEs and their association with mental health and offending in African and South African offenders have supported the finding that women’s pathways to offending are associated with prior sexual and physical victimisation, adverse life events, substance abuse, and mental health problems.^18,19,20,21,22^ Adverse childhood experiences are also associated with recidivism;^23^ hence, identification of, and intervention efforts directed at management of ACEs, are crucial.

### Adverse childhood experiences and violent offending

According to Widom’s ‘cycle of violence theory’ violent victimisation, particularly physical abuse inflicted upon children by their carers or parents, increases the risk of later violent behaviour.^24^ In addition, children who suffer neglect are also more likely to develop subsequent violent criminal behaviour.^24^ The ACE study revealed the cumulative negative impact of ACEs on health in adulthood, as well as an elevated likelihood for the perpetration of aggressive acts with cumulative ACEs.^25^

Adverse childhood experiences are indeed linked to violent offending in both genders. However, violent offending particularly interpersonal violence in females has been linked to defensive reactions related to chronic past abuse, both in South Africa^26^ and internationally.^27^

A meta-analysis investigating the cycle of violence after exposure to childhood maltreatment among women, in the general and prison populations, found a significant, albeit small, positive association between ACEs and a wide array of violent behaviours.^28^ A Swiss study comparing male and female violent offenders, reported that violent female offenders were more likely to have experienced ACEs, such as sexual trauma, as opposed to male offenders.^29^ A prospective study in the US reported that females with a history of any childhood abuse or neglect were at significantly higher risk of arrest for violence compared to the control group.^30^ Hence, there is a significant body of literature demonstrating a link between ACEs and violent offending among female inmates.

### Adverse childhood experiences and human immunodeficiency virus

Human immunodeficiency virus remains a major public health burden; hence, understanding the role of risk factors such as ACEs is essential.^31^ Adverse childhood experiences are increasingly being recognised as risk factors for human immunodeficiency virus (HIV) and HIV-related diseases,^31^ and are prevalent among people living with HIV and AIDS (PLWHA).^32^ Adverse childhood experiences contribute to risky behaviours such as hazardous drinking, illicit drug use, risky sexual behaviours, and they increase the likelihood of being a victim of intimate partner violence later in life.^33,34,35,36,37,38,39^ South Africa (SA) has the largest HIV epidemic in the world.^40^ KwaZulu-Natal (KZN) has the second highest population in the country and carries the highest burden of HIV, with women being disproportionately affected.^40^ The first phase of the larger study in which we measured the prevalence of mental illness, borderline personality disorder (BPD) and antisocial personality disorder (ASPD) using the Structured Clinical Interview for the Diagnostic and Statistical Manual of Mental Disorders 5th edition-Research Version (SCID 5-RV), and the prevalence of HIV using prison medical records, reported a 64.3% prevalence of HIV among the female inmates at a correctional centre in KZN.^41^ Therefore, it would be important to determine the prevalence of ACEs in this HIV-dense female inmate population.

The literature suggests complex associations between ACEs, mental health pathology, and HIV, but most of the data are from high-income settings and predominantly male offender populations.^42^ Female offenders are a highly vulnerable population who are often living with the dual burden of HIV and mental illness, but have received limited attention.^43^ Hence, there is a need to better understand the prevalence and type of adverse childhood experiences and their associations with later mental illness, personality disorders, violent offending and HIV among female inmates in a LMIC setting. This will help us formulate more appropriate prevention and treatment interventions for LMIC settings.

To date, there is no quantitative literature on childhood adversities and its associations in female inmate populations in SA. Hence, this study aims to fill this gap by describing the prevalence of lifetime trauma (including ACEs), and investigating relationships between cumulative childhood adversities and mental illness, personality disorders, violent offending and HIV, among female inmates in a LMIC, HIV-dense, South African setting. This study was conducted in Durban, KZN, as it is a largely under-researched area geographically.

## Research methods and design

### Study design

This was a cross-sectional, descriptive study among female inmates. The findings reported here form part of the first phase of a two-phased, mixed methods, sequential explanatory design study which was conducted at a correctional centre in Durban, KZN, SA. Phase one of the study measured the prevalence of mental illness, BPD and ASPD using the SCID 5-RV.^44^ The prevalence of childhood and adult trauma was also measured. Human immunodeficiency virus data was confirmed using prison medical records. The nature of the charge (violent or non-violent) was obtained from correctional services records. This manuscript reports on the trauma component of the first-phase findings.

### Study population and sampling

This study was conducted at the largest correctional centre in Durban, KZN. Male and female inmates are accommodated at this correctional centre; the majority of whom are black and isiZulu speaking.

A sample size of 126 was calculated by the statistician to be an adequate sample size. The first author informed and invited all 349 female inmates to participate in the study before the study commenced. A random sample of 126 was then drawn from all the women who had agreed to participate. This included 96 sentenced offenders (SOs) and 30 remand detainees (RDs). To be eligible for phase one, participants had to be 18 years of age or older, be able to provide written informed consent, and be either English or isiZulu speaking. Those who lacked capacity to provide informed consent were excluded.

### Data collection

Data were collected from August 2019 to November 2019. The first author, who is a forensic psychiatrist, interviewed all the participants. An English-isiZulu translator was employed to assist with participants who were not fluent in English, who accounted for a minority of the total sample. The same research assistant was used to interview all participants who required translation from isiZulu to English and vice versa. She is a qualified social worker with previous research experience in psychiatry, as well as a research training qualification from the National Research Foundation. Her first language is isiZulu. During interviews with participants who were not fluent in English, questions were asked in English by the first author; they were then translated by the research assistant into isiZulu. Answers were given by the participants in isiZulu and then translated back into English to the first author for documenting. The research assistant was instructed not to interpret answers but to provide translation verbatim.

#### Instruments

**Socio-demographic, clinical and forensic questionnaire:** A socio-demographic, clinical and forensic questionnaire, based on a review of the literature, was administered by the first author in English and isiZulu, with the aid of a translator. The clinical component contained questions about, inter alia, HIV status, physical, sexual and emotional abuse experienced in adulthood (after age 18). Details regarding the types of physical injuries sustained and medical intervention sought were also documented.

### World Health Organization Adverse Childhood Experiences-International Questionnaire (WHO ACE-IQ)

The WHO ACE-IQ was used to measure childhood adverse experiences.^45^ It has been used in a previous study on females in KZN, SA,^10^ and it has been validated in a Nigerian prison population.^46^ The Nigerian study found that the ACE-IQ and Child Trauma Questionnaire (CTQ) had concurrent validity, and that the ACE-IQ is a reliable and valid index of adverse childhood experiences in the prison population.^46^ The ACE-IQ measures 13 categories of childhood adverse experiences, which are listed in Table 1.

**TABLE 1:** Prevalence of Adverse childhood experiences using the Adverse Childhood Experiences – International Questionnaire binary and frequency versions as reported by the 126 participants.

ACE category	Binary version		Frequency version	
	*N*	%	*N*	%
Physical abuse	110	87.3	78	61.9
Emotional abuse	118	93.7	97	77.0
Contact sexual abuse	51	40.5	51	40.5
Alcohol and/or drug abuser in household	71	56.3	71	56.3
Incarcerated household member	27	21.4	27	21.4
Household member depressed/mentally ill/institutionalised or suicidal	28	22.2	28	22.2
Household member treated violently	113	89.7	102	81.0
One or no parents/parental separation/divorce	74	58.7	74	58.7
Emotional neglect	35	27.8	43	34.1
Physical neglect	25	19.8	16	12.7
Bullying	80	63.5	56	44.4
Community violence	100	79.4	63	50.0
Collective violence	63	50.0	63	50.0

ACE, Adverse Childhood Experiences.

There are two scoring systems: the binary and frequency versions. The binary version is a ‘yes’ or ‘no’ rating system that measures the presence or absence of each adversity, while the frequency version is a measure of the severity of each adversity. Both scales are scored out of 13. The two categories of community violence and collective violence (which are not included in the 10 item CDC- Kaiser Permanente ACE scale) are particularly relevant for LMICs like SA, where these experiences may be more prevalent.^47^ Bullying and parental death are additional adverse events which are included in the WHO ACE-IQ, but are not part of the original CDC-Kaiser Permanente ACE scale.

The main outcome measures were mental illness, ASPD, BPD, HIV infection, and offending behaviour (either violent or non-violent).

### Data collation

The first author captured the data online using Research Electronic Data Capture (REDCap) which is a browser-based, meta-data-driven electronic data capture software solution and workflow methodology for designing clinical and translational research databases.^48,49^

### Data analysis

International Business Machines Statistical Package for the Social Sciences (SPSS IBM-International Business Machines, Armonk, New York, United States) version 26 was used to analyse the data. Frequency tables with percentages, as well as graphs were used to describe categorical variables. Logistic regression models were used to estimate the odds ratio (OR) and 95% confidence interval (CI) associated with the odds of being exposed to ACE scores ≥4 compared to ACE scores <4 for each mental illness, ASPD, BPD, violent offending and HIV (*p* < 0.05).

The literature suggests that exposure to cumulative ACEs is more significant than exposure to a single ACE.^50^ Felitti and colleagues reported that individuals who had experienced four or more categories of childhood exposure, compared to those who had no exposure, had a 4- to 12-fold increased risk for alcoholism, drug abuse, depression, and suicide attempt; a 2- to 4-fold increase in smoking, poor self-rated health, ≥50 sexual intercourse partners, and sexually transmitted diseases; and a 1.4- to 1.6-fold increase in physical inactivity and severe obesity.^50^ Other studies have also used 4 as a cut-off; hence, we elected to use this cut-off to facilitate comparison with the literature.^50^ However, the caveat being that our study used the WHO ACE-IQ (13 item scale) while the other studies used the CDC-ACE scale (10-item scale). Hence, direct comparison may not be possible.

### Ethical considerations

Prisoners are considered a vulnerable population. Hence, strict ethical principles were adhered to throughout the study. Approval for the study was obtained from the University of the Witwatersrand Human Research Ethics Committee (certificate number M181026) and from the Department of Correctional Services (DCS). All participants consented in writing after being fully informed. Informed consent was also obtained to gain access to participants’ prison medical records to confirm their HIV status. A distress protocol was in place if needed. Participants who required urgent intervention after the interview were referred, with their consent, to the prison doctor or psychologist as deemed necessary. The first author emphasised to participants that she was an independent researcher not affiliated with the DCS and that the DCS would not have access to the data. They were also informed that participation in the study would not affect their criminal proceedings. Participants were also informed that the data collected would remain confidential and their anonymity would be ensured. Data were electronically captured using unique participant identification numbers, were stored (password protected) and were only accessible by the first author. Participants were also made aware that they could withdraw from the study at any stage if they so wished, without affecting their care at DCS. A hygiene pack (consisting of basic sanitary items) to the value of R60 was given to each participant for their time.

## Results

The socio-demographic, forensic profile and mental illnesses of the 126 participants have been published elsewhere.^41^

### Prevalence of individual adverse childhood experiences

Table 1 summarises the prevalence of ACE scores reported by the participants using the binary and frequency versions.

The three most common adverse experiences on both the binary and frequency versions were: physical abuse, emotional abuse and witnessing a household member treated violently. Experiencing sexual abuse, community violence, collective violence, and living with a substance-abusing household member were also prevalent. While 77% (*n* = 98) of the participants had more than six ACEs on the binary version, and 61.1% (*n* = 77) had more than six ACEs on the frequency version. Notably, 38.9% (*n* = 49) and 32.5% (*n* = 41) had experienced both physical and sexual abuse on the binary version and frequency version, respectively.

### Prevalence of cumulative adverse childhood experiences

**FIGURE 1:**
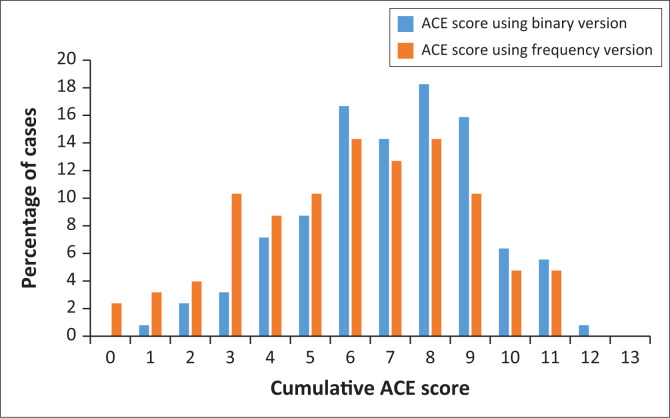
Prevalence of the cumulative adverse childhood experience scores using the binary and frequency versions.

Figure 1 indicates the frequency of the cumulative number of ACEs experienced by participants using the binary and frequency versions of the WHO ACE-IQ. The main finding is that 100% of participants reported experiencing at least one ACE using the binary version (which measures the presence of ACEs). Over 97% of participants reported experiencing ACEs using the frequency version (which measures the severity of the ACEs experienced). The most frequent cumulative number of ACEs experienced were 8 and 6, using both the binary and frequency versions. Over 93% (*n* = 118) reported four or more ACEs with the binary version and 80.2% (*n* = 101) with the frequency version.

### Association between cumulative adverse childhood experiences, mental illnesses, personality disorders and human immunodeficiency virus

Table 2 shows the unadjusted OR of having a *The Diagnostic and Statistical Manual of Mental Disorders, Fifth Edition*, (DSM-5) disorder or HIV infection for ACE ≥4 versus ACE <4 using both binary and frequency versions.

**TABLE 2:** Association of adverse childhood experiences with DSM-5 disorders and human immunodeficiency virus using binary and frequency versions.

Lifetime disorders	ACE Binary score ≥ 4 versus < 4		ACE Frequency score ≥ 4 versus < 4	
	OR	95% CI	OR	95% CI
Psychotic disorders	Cannot be computed because of 0 instances of those with ACE binary score <4 having psychotic disorder	-	Crude: 0.47 Adjusted: 0.226	0.08–2.75 0.026–1.930
Depressive disorders	Crude: 0.36 Adjusted: 0.878	0.04–2.74 0.076–10.203	Crude: 2.27 Adjusted: 3.272	0.92–5.61 0.997–10.742
PTSD	Crude: 6.77 Adjusted: Cannot be computed	0.81–56.72	Crude: 6.28 Adjusted: 9.167	2.01–19.60 2.243–37.467
ADHD (current)	Crude: 0.72 Adjusted: Cannot be computed	0.08–6.40	Crude: 1.26 Adjusted: 6.857	0.26–6.17 0.466–100.894
AUD	Crude: 3.00 Adjusted: 1.370	0.58–15.47 0.168–11.183	Crude: 6.80 Adjusted: 8.373	2.18–21.25 2.153–32.559
SUD	Crude: 3.68 Adjusted: 1.494	0.71–18.98 0.175–12.787	Crude: 3.76 Adjusted: 3.993	1.44–9.82 1.150–13.864
BPD	Crude: 1.54 Adjusted: 1.882	0.30–7.97 0.199 – 17.797	Crude: 3.17 Adjusted: 4.752	1.01–9.93 1.175 – 29.220
ASPD	Crude: 1.26 Adjusted: 0.357	0.15–10.87 0.021–6.153	Crude: 2.33 Adjusted: 2.831	0.50–10.81 0.385 – 20.840
HIV	Crude: 3.25 Adjusted: 5.702	0.74–14.30 0.656–49.554	Crude: 5.54 Adjusted: 5.367	2.15–14.28 1.507–19.111

HIV, human immunodeficiency virus; ACE, Adverse Childhood Experience; OR, odds ratio; CI, confidence interval; PTSD, post-traumatic stress disorder; ADHD, attention-deficit/hyperactivity disorder; AUD, alcohol use disorders; SUD, substance use disorders; BPD, borderline personality disorder; ASPD, antisocial personality disorder.

Univariate logistic regression models used.

Adjusted models were adjusted for age, education, income and population group.

Calculations could not be computed for bipolar disorder because there were zero instances of those with an ACE score <4 having bipolar disorder, as well as only one participant having bipolar disorder in the total sample.

There were insufficient cases with scores <4 (*n* = 8) for ACEs on the binary version to reliably report and association. However, several associations emerged from the cumulative ACE scores on the frequency version. On the frequency version, those with ACE scores ≥4 were 6.28 times more likely to have PTSD; 6.8 times more likely to have an alcohol use disorders (AUD); 3.8 times more likely to have a substance use disorders (SUD); 3.17 times more likely to have a BPD; and 5.54 times more likely to have HIV infection than those with ACE scores <4. Adjusting the ORs (on the frequency version) for age, race, income and level of education did not change the conclusions from the unadjusted values; however, in many instances, it inflated the OR and resulted in wider CIs.

### Association between adverse childhood experiences and violent offending

There were no significant associations between cumulative ACE scores and violent offending using logistic regression models on the binary and frequency versions. Those with an ACE score ≥4 on the binary version were 1.62 times (95% CI 0.37–6.81) more likely than those with ACE score <4 to have committed a violent crime, but the CIs overlapped with 1 thus, the risk was not significantly higher. Notably, there were only eight participants with an ACE score <4 on the binary version. Adverse childhood experiences scores ≥4 on the frequency version also did not confer higher odds of committing a violent crime, OR 0.86 (CI 0.35 to -2.13). In those with ACE scores <4 on the frequency version, 64% (*n* = 16/25) committed violent crimes, while in the group with ACE scores ≥4, 60% (*n* = 61/101) committed violent crimes.

### Prevalence of adult physical, sexual and emotional abuse

Overall, 70.6% (*n* = 89) reported physical abuse; 20.6% (*n* = 26) reported sexual assault or rape; and 80.2% (*n* = 101) reported being emotionally abused as adults. Sixty-one per cent (*n* = 78) of participants sustained soft tissue injuries from interpersonal violence as adults; 11.9% (*n* = 15) fractures; 23.0% (*n* = 29) stab wounds; and 1.6% (*n* = 2) gunshot wounds. Forty-one per cent (*n* = 52) of participants reported seeking medical attention for injuries they had sustained from interpersonal violence as an adult.

## Discussion

Key findings of this study were the high prevalence of ACEs on both the binary and frequency versions, thus confirming not just the presence of adversities, but also their severity. There were also significant associations between cumulative ACEs (using the frequency version) and PTSD, AUD, SUD, BPD and HIV infection among female inmates in Durban, South Africa.

Jewkes and colleagues, although they did not use the full description of ACEs in their study, reported that 54.7% of rural South African women experienced emotional abuse; while 41.6% experienced emotional neglect; and 39.1% experienced sexual abuse before the age of 18.^51^ Manyema and Richter’s study in the South African general population (the Soweto Birth to Twenty Plus cohort) found lower rates of ACEs among women compared to our study.^12^ The most common ACEs reported in their study were parental divorce or separation (46%), emotional abuse (30%), and emotional neglect (31%). Two items included in their study, in addition to the CDC-Kaiser Permanente ACE scale, were household chronic illness and unemployment, which are relevant to the LMIC South African context, and measured 27% and 41%, respectively. In their study, 88% reported at least one ACE while 34% of women had four or more ACEs^12^ whereas in our study, 100% reported at least one ACE on the binary version (97.6% on the frequency version) and 93.7% reported four or more ACEs with the binary version (80.2% with the frequency version). This illustrates that the female incarcerated population in SA is afflicted with a heavier burden, both in terms of the number and severity of childhood adversities, compared to females in the general population.

This is also consistent with international general population rates of ACEs among high, middle and low-income countries as measured by the World Mental Health Survey (WMHS), which found that rates for physical abuse ranged from 5.3% to 10.8%, sexual abuse ranged from 0.6% to 2.4%, and neglect ranged from 3.6% to 5.2%.^52^ Overall, 61.5% had at least one ACE, while 7.0% had four or more ACEs. All 12 ACEs were significantly associated with DSM-5 disorders assessed in the WMHS.^52^ Prevalence rates of experiencing four or more ACEs in the general population ranging from 1% to 32%, were found in a more recent systematic review.^9^ Only studies from high- and middle-income countries were included in this review.^9^ The highest prevalence included in this systematic review was from a middle-income country,^9^ which is still substantially less than our findings.

Significantly, a cumulative Kaiser Permanente-CDC ACE score of six or more places individuals at increased risk of dying 20 years younger, of diseases commonly diagnosed in the primary care setting, compared with individuals without exposure to six or more ACEs.^53^ Even though our study used the WHO ACE-IQ, 10 items overlapped with the Kaiser Permanente-CDC ACE. Thus, the majority of women in our study who had six or more ACEs may likely fall into this high risk category for premature death, which is a cause for serious concern.

All studies in the systematic review by Bowen and colleagues also demonstrated substantially higher rates of ACEs in prisoners compared to community samples.^17^ Studies have reported that a large proportion of female prisoners have experienced childhood physical abuse, childhood sexual abuse, or both, which is consistent with our findings.^54,55^ Unfortunately, as Kennedy and colleagues (US) have highlighted, prison mental health services have not been designed to address this huge burden of childhood victimisation,^56,57^ which is likely to be the same in South African correctional centres.

Extant literature has reported that rather than occurring as singular experiences, individuals often experience multiple ACEs.^7,9^ This finding is consistent with our study. Furthermore, cumulative exposures to multiple forms of trauma highlight the detrimental impact of the trauma.^58,59,60^ A systematic review and meta-analysis, consisting of data from predominantly high-income countries, found that 45.5% of participants in the studies reviewed had at least one ACE.^61^ Similar to the ACE study, the review concluded that there was a graded relationship between ACEs and psychosocial or behavioural outcomes, that is, the more ACEs one had, the higher the likelihood of negative outcomes, which included tobacco use, alcohol problems, illicit drugs, obesity, risky sexual behaviour, depressed mood, suicidal ideation, being a victim of violence, psychological distress, hallucinations, anxiety or panic, and poor health or quality of life.^61^

The systematic review of prisoners by Bowen and colleagues further corroborated the association between childhood adverse events and the presence, number or severity of mental illnesses in adults. Specifically in female prisoners, they found that cumulative trauma was associated with PTSD, anxiety, mood disorders, alcohol and substance abuse, BPD, ASPD, and psychopathy. The majority of these findings are consistent with those of our study.^17^ Our study highlights the risk that cumulative ACEs impose on female inmates and adds support to the dose-response relationship reported in previous studies.

With respect to psychotic disorders, Varese and colleagues in their 2012 meta-analysis, reported associations between individual categories of childhood abuse (physical, sexual, emotional abuse and neglect) and psychosis.^62^ In addition, Kennedy and colleagues concluded that multi-victimisation in childhood is a risk factor for the development of psychosis among female prisoners.^56^ Owing to the small proportion of women with psychosis in our study, an association between childhood victimisation and psychosis could not be established.

No association was found between cumulative ACEs and violent offending in this study using both the frequency and binary versions. Previous studies have found associations between individual ACEs and violent offending, but investigations of associations between individual ACEs and violent offending were beyond the scope of this manuscript. This will be a focus of future publications. Another likely reason to explain the lack of association between cumulative ACEs and violent offending is that this study had a limited sample size.

This study found that women with ≥4 ACEs were 5.54 times more likely to be HIV infected than those with <4 ACEs on the frequency version. This is consistent with the literature which has reported that ACEs are more common in PLWHA.^63,64^ Adverse childhood experiences may have an effect on sexual risk behaviours, which increases the risk of sexually transmitted diseases including HIV.^65^ Among PLWHA, trauma is associated with mental illness, poor medication adherence, poor quality of life, faster disease progression and higher mortality rates.^66,67,68,69,70,71,72^ Even PLWHA who are virally suppressed on antiretroviral treatment, are disproportionately affected with mental health problems.^73,74^

### Limitations

Participants in this study were from a single correctional centre in SA, and this may limit generalisability of the findings. Random sampling was only conducted on those who agreed to participate; thus, the possibility of sampling bias does exist. The sample size was limited; hence, the study was underpowered for showing associations between risk factors and mental illnesses. Furthermore, sensitive information was elicited via face-to-face interviews and therefore, social desirability bias may have contributed to under- or over-reporting. In addition, many studies in the literature used the Kaiser Permanente’s 10-item ACE score, while our study used the 13-item WHO ACE-IQ score and thus direct comparison may not be possible. An isiZulu-English translator was used for interviews with participants who were not fluent in English, which may have influenced the findings. Finally, this was a cross-sectional study; hence, no causal inferences can be made.

## Conclusion

This study contributes to the emerging literature on ACEs and their associations among incarcerated populations, particularly in a LMIC setting. It found that female inmates in Durban, SA, are a highly traumatised population as evidenced by the high rate of adversities experienced during childhood and adulthood. It also found an association between cumulative ACEs and mental illnesses such as PTSD, SUD, AUD, BPD, as well as between ACEs and HIV. This suggests the need for early screening and intervention for those inmates with ACEs, as well as comprehensive mental health care delivery which includes trauma and HIV care within mental health services. It is imperative for service providers in correctional facilities to adopt a trauma-informed lens so that they can understand the prevalence of trauma and its lasting deleterious impact on an inmate’s psychological health. The authors therefore recommend that similar studies be undertaken at other correctional centres in SA, and in other LMICs so that findings may be compared. More importantly, our findings may serve as an evidence base to enable the formulation and implementation of gender-sensitive, trauma-informed policies and trauma-focussed interventions, which may benefit these women by improving their mental health and re-offending outcomes. Future studies should also take additional childhood adversities into account, such as the effects of adverse socio-economic circumstances.
